# Factors related to the physician and the employer influencing successful return to work in Korea: results from the first panel study of workers’ compensation insurance (PSWCI)

**DOI:** 10.1186/s40557-015-0076-x

**Published:** 2015-12-11

**Authors:** Wanhyung Lee, Jin-Ha Yoon, Jaehoon Roh, Yeong-Kwang Kim, Hongdeok Seok, June-Hee Lee, Jong-Uk Won

**Affiliations:** Department of Preventive Medicine, The Institute for Occupational Health, Yonsei University College of Medicine, 50, Yonsei-ro, Seodaemun-gu, 120-749 Seoul, Korea; Graduate School of Public Health, Yonsei University College of Medicine, Seoul, Korea; Incheon Worker’s Health Center, Incheon, Korea; Department of Preventive Medicine, Yonsei University College of Medicine, Seoul, Korea

**Keywords:** Return to work, Industrial accident, Occupationally injured worker, Workers’ compensation insurance

## Abstract

**Objectives:**

This study aims to investigate associated factors including the physician and the employer of successful return to work (RTW) in occupationally injured workers.

**Methods:**

This study is based on the first panel study of workers’ compensation insurance (PSWCI), published in June 2014. The PSWCI is a sample survey of occupationally injured workers who completed medical care in 2012 (89,921 people). A total of 2000 subjects were sampled based on sex, age, nine metropolitan-based regions, disability ratings, duration of rehabilitation, and whether vocational rehabilitation service was used. We divided the study population into two groups: return to work (RTW) group (job retention, reemployment, unpaid family worker, and self-employment), and non-RTW group (joblessness and economical inactivity). The odds ratios (ORs) and 95 % confidence intervals (CI) related to differences in basic characteristics, part of physician and employer-related factors between those who succeeded to RTW and those who did not were measured using multivariable logistic regression model.

**Results:**

The success of RTW is 70.6 % (*n* = 1412) among participants. The ORs (95 % CI) of the participants belonging to RTW who received periodic recovery assessment from the medical care institution and the physician are 1.51 (1.07–2.13). The ORs (95 % CI) are 1.68 (1.05–2.69) for the RTW group who received work ability assessment and referral for vocational return. When the employer maintains the relationship with the occupationally injured worker, the worker has 1.39 times higher odds (95 % CI: 1.41–2.26) of the RTW group compared to the non-RTW group.

**Conclusions:**

The physician and the employer have a significant impact on the RTW.

## Introduction

Occupationally injured workers suffer from physical pain, and they worry about the possibility of full recovery; moreover, they are anxious about the future [1, 2]. Such fears are mainly caused by concerns about their participation in future economic activity, which is closely associated with their own welfare as well as that of their family and social activity [3]. Failure to return to work (RTW) increases the risk of social isolation, reduces meaningful activity and may make the worker doubting his/her own competence [4]. In addition, If a worker cannot success to RTW due to an industrial accident, the worker and his/her family would fall to a low socio-economic status, which would cause problems, thereby deepening the polarization in society [5].

Successful rehabilitation is achieved when an occupationally injured worker recovers through medical care and rehabilitation services (primary recovery) and when secondary recovery is made through the RTW. And the RTW can be a role to ensure social justice and social harmony through reducing individual and national financial burden of industrial accident [6, 7]. Therefore, RTW is a good index to measure the successful rehabilitation of an occupationally injured worker [8–10].

The RTW of occupationally injured workers needs to be analyzed using a multi-dimensional approach because various factors such as personal characteristics, industrial factors, role of the physician, and the relationship with employers could be associated with the occupationally injured workers’ RTW [11, 12]. Especially, role of the physician and association with the employer have pivotal roles in RTW of occupationally injured workers.

The physician is particularly instrumental in the process of RTW after industrial accident. For example, the physician can be helpful to RTW through an understanding of the job’s demands by detailed discussions with the occupationally injured workers [13].

Attitude and role of the employer are also well known for helping RTW in occupationally injured workers. The employer should communicate more frequently with workers who suffered industrial accident during medical care and rehabilitation as well as hold follow-up meetings more often as this is associated with a faster RTW in occupationally injured workers [14, 15].

In order to get a better understanding of process to RTW, it is necessary to identify factors focused on the physician and the employer that are associated with successful RTW in occupationally injured workers. Therefore, this study analyzed the factors of the RTW including role of the physician and the employer in Korea and its effect on the RTW using data from the first panel study of workers’ compensation insurance (PSWCI).

## Materials and methods

### Source of study population

Data from the first PSWCI, published in June 2014, were used for all the data analyses in this study. The PSWCI objectively identified the effect of the insurance policy system on occupationally injured workers. This policy is managed by the Korea Workers’ Compensation and Welfare Service (KCOMWEL) for providing appropriate services and aiding RTW. The PSWCI is a sample survey of injured workers who terminated medical care in 2012. There were 89,921 people in the target population of the survey. The study population was limited to 82,493 people by excluding people whose address was unknown (73 people), foreigners, or residents of Jeju-island, or nonuser of rehabilitation services (7355 people). Finally, 2000 subjects were sampled based on sex, age, nine metropolitan-based regions, disability ratings, duration of rehabilitation, and whether rehabilitation services were used. To ensure objective data collection, the PSWCI conducts computer-assisted 1:1 personal interviews.

### Economic activity status

The subjects are divided into two groups according to economic activity status. The RTW group is further divided into four sub-groups: job retention, reemployment, unpaid family worker, and self-employment. The job retention group includes workers returning to the workplace where the industrial accident occurred. Reemployment refers to the situation where a worker takes up another job after the industrial accident. If the time invested for helping the family and relatives of the injured worker exceeds an average of 18 h per week (over 3–4 h/day), the classification is under the unpaid family worker sub-group. The self-employment sub-group is characterized by involvement in work related to private business, freelancing, shops, and restaurants.

The non-RTW group is divided into two sub-groups: joblessness and economical inactivity. Joblessness refers to a situation where a worker has been approached for an income-based job at least once, and he/she has confirmed willingness to work. Economical inactivity means that the worker has not made an effort to find a job in the last 4 weeks or has stated that he/she cannot work even if there is a suitable position available during in the last 1 week.

### Occupational characteristics

Occupational category was re-grouped as manual, non-manual, and other from the 10 categories defined by the Korea Standard Occupation Classification. Individuals in agriculture, forestry, fishery, engineering, assembling, technical work, and manual labor were classified as manual workers. Managers, experts and related workers, and office workers were classified as non-manual workers. Individuals with sales, services, and soldier were classified as other.

The number of workers is used to determine the size of the enterprise: less than five workers, between 5 and 9 workers, between 10 and 29 workers, and more than 30 workers. To assess the working conditions, the following factors were investigated: employment status (regular, temporary, daily, or self-employed), type of work (full-time or part-time), shift work, and whether an employment contract had been signed. Part-time workers included those with a side job, those employed hourly, and those whose working time was at least 1 h shorter than that of the other workers in the same company. The employment contract refers to the labor contract that describes the detailed conditions of service including payment, period of work, and so on.

### Factors related to the physician and the employer

The analysis considers two different aspects in the PCWCI: part of the physician and the employer-related factors. The PSWCI question related to part of the physician “Did the physician discuss the injured area, degree of injury, and estimated treatment period in detail?” is represented as “detailed explanation of medical care” in this study. The question “Did the medical care institution and the physician assess the degree of periodic recovery for you?” is represented as “regular assessment of recovery”. The respondents were of the opinion that the adequacy of the treatment period indicates “the appropriateness of the treatment duration.” The question “Have you ever received any counseling from the physician for returning to workplace as part of the treatment process?” is represented as “counseling about returning to work”. The question “Have you ever received any work capacity evaluation and referral for vocational return provided by KCOMWEL?” is represented as “work capacity assessment and referral for vocational return”.

Regarding the employer related aspect, the question “Do you continue to maintain your relationship (visits, telephone calls, etc.) with the employer or personnel of the department of human resources (HR) at the workplace during convalescence after the industrial accident?” is represented as “maintenance of relation”. Similarly, for the question “Are any facilities provided by the employer during convalescence after the industrial accident?” the subjects responded positively if they received any goodwill instead of benefits such as the cost of outpatient treatment or salary payment. This is represented as “provided convenience during recovery”.

The PSWCI response options for the questions related to additional compensation from the physician and the employer of RTW consisted of five alternatives: “very dissatisfied,” “dissatisfied,” “moderate,” “satisfied,” and “very satisfied.” However, considering the subjectivity of the responses and a significant amount of dissatisfaction, the analysis includes “very dissatisfied to dissatisfied: low,” “normal: moderate,” and “satisfied to very satisfied: high”.

### Other covariates

Among the general characteristics of an occupationally injured worker, the level of education was divided into eight groups in the industrial accident panel investigation. However, to enhance the efficiency and understanding of the analysis, it was divided into three groups in this study based on the final graduation level: middle school, high school, and higher than college. Household income increases as it moves from the first to the fourth quartile. The monthly average household income is divided into four slabs: below ₩ (Korean Won) 1 million (approximately 1100 Korean won = 1 U.S. dollar) in the first quartile, between ₩ 1 and 1.8 million in the second quartile, between ₩ 1.8 and 2.9 million in the third quartile, and above ₩ 2.8 million in the fourth quartile. Smoking habit and alcohol consumption were grouped into never, past, and current. “Current” was defined as having experience smoking or drinking alcohol at the time of the survey, those who no longer smoked or drunk but smoked or drunk in the past as “past,” and those who never smoked or drunk as “never.” The registered disabled people were investigated based on the question “Did you register your disability from occupationally accidents in a local government?” Those who had registered their disability were evaluated in terms of the grade of the handicap, from maximum 1 (worse) to minimum 14 (better).

### Statistical analysis

The SAS statistical package version 9.3 is used in this study. The chi-square and student’s *t*-tests are applied to obtain the statistical difference between the RTW group and the non-RTW group. The odds ratios (ORs) and the 95 % confidence intervals (CI) are calculated through multivariable logistic regression analysis to identify the impact on the RTW. When the *p*-value is less than 0.05 for all the statistics results, it is considered statistically significant.

## Results

### Present condition and baseline characteristics

The present conditions of the RTW and the non-RTW group are shown in Table 1. Of the 2000 people considered in this research, 1412 people belong to the RTW group (70.6 %). The remaining 588 people (29.4 %) belong to the non-RTW group. The results show that job retention is the most common trend in the RTW, with this sub-group accounting for 695 people (34.8 %). The reemployment sub-group follows next, with 642 people (32.1 %). In the non-RTW group, most of the people (443 people, corresponding to 22.2 %) are categorized under economically inactivity, and the remaining 145 people are classified under joblessness.

**Table 1:** Dispersion of study population according to economic activity status

	Number of people	Percentage
Return to work group		
Job retention	695	34.8
Reemployment	642	32.1
Self-employed	67	3.4
Unpaid family worker	8	0.4
Subtotal	1412	70.6
Non-return to work group		
Joblessness	145	7.3
Economically inactivity	443	22.2
Subtotal	588	29.4
Total	2000	100.0

The general characteristics of the RTW group and non-RTW group are shown in Table 2. The proportion of women in the non-RTW group is observed to be relatively higher (20.7 %) than that in the RTW group (13.6 %), and it is statistically significant. In terms of the age group, most of the respondents were aged 50–59 (33.7 and 33.8 %) in both the groups. There was a difference in the educational status of the people in the RWT group and those in the non-RTW group. The proportion of the college graduates or people with higher education in the RWT group was 20.3 %, while the non-RTW group was 11.7 % (*p* <0.0001). The income levels in the both the RTW and the non-RTW groups are relatively higher in the first quartile. Smoking and alcohol consumption are relatively low in the non-RTW group. In terms of registered disability, 147 people registered under this category; this figure accounts for 7.4 % of the total respondents. Among all the respondents with disabilities, 81 people (55.1 %) belong to the non-RTW group. The average impairment rating of a registered disabled person in the non-RTW is 4.19, which represents a worse degree of impairment compared to that in the RTW group, which is 5.04 (*p* = 0.0016).

**Table 2:** Basic characteristics of study population according to economic activity status

	Return to work group	Non- return to work group	*p*-value
	*N* = 1412 (% = 70.6)	*N* = 588 (% = 29.4)	
Sex			<0.0001
Men	1220 (86.4)	466 (79.3)	
Women	192 (13.6)	122 (20.7)	
Age (years)			< 0.0001
<40	317 (22.9)	95 (16.7)	
40 ~ 49	408 (29.5)	115 (20.2)	
50 ~ 59	467 (33.7)	193 (33.8)	
≥60	193 (13.9)	167 (29.3)	
Education			< 0.0001
Middle school	456 (32.3)	279 (47.5)	
High school	669 (47.4)	240 (40.8)	
College or higher education	287 (20.3)	69 (11.7)	
Household income			< 0.0001
1^st^ quartile	1016 (72.0)	383 (65.1)	
2^nd^ quartile	262 (18.6)	136 (23.1)	
3^rd^ quartile	91 (6.4)	48 (8.2)	
4^th^ quartile	43 (3.0)	21 (3.6)	
Smoking			0.0017
Current	722 (51.1)	253 (43.0)	
Past	278 (19.7)	132 (22.5)	
Never	412 (29.2)	203 (34.5)	
Alcohol consumption			< 0.0001
Current	1076 (76.2)	365 (62.1)	
Past	91 (36.4)	86 (14.6)	
Never	245 (17.4)	137 (23.3)	
Registered as disabled			< 0.0001
Yes	66 (4.7)	81 (13.8)	
No	1346 (95.3)	507 (86.2)	
Mean of disability rating^a^	5.04 ± 1.16	4.19 ± 1.70	0.0016

^a^in terms of registered disabled people

### Occupational characteristics

The occupational characteristics of the RTW group and the non-RTW group are summarized in Table 3. The present conditions of these two groups are identified according to the occupational categories. The manual workers are the most common among the occupationally injured workers in both these groups: 1161 (82.2 %) people from the RTW group and 513 (87.2 %) people from the non-RTW group belonged to this category. Regarding the size of the enterprise (number of workers), 45.8 % of the RTW group and 51.7 % of the non-RTW group worked in enterprises with fewer than 10 workers, which is statistically significant (*p* = 0.0114). In the RTW group, there were more regular workers than workers in the temporary or daily categories. With respect to the difference in distribution according to the type of workers, the proportion of temporary and daily workers in the non-RTW group appears to be high (*p* <0.0001). There were more full-time workers than part-time workers in the RTW and non-RTW groups in terms of working hours (96.7 and 94.7 %, respectively). The opposite proportional rate is observed between the RTW group and the non-RTW group (51.4 and 42.5 %, respectively) if employment is offered under a written labor contract (*p* = 0.0003). There are no significant statistical difference between the RTW and the non-RTW groups, as the monthly average number of working days are 21.81 (±5.09) and 21.79 (±5.92) days, and the daily average working hours are 9.40 (±2.47) and 9.51 (±2.50) hours. (*p* = 0.9463, 0.3300).

**Table 3:** Occupational characteristics of study population according to economic activity status

	Return to work group	Non-return to work group	*p*-value
	*N* = 1412 (% = 70.6)	*N* = 588 (% = 29.4)	
Occupational category			0.0241
Non-manual	151 (10.7)	35 (36.0)	
Manual	1161 (82.2)	513 (87.2)	
Other	100 (37.1)	40 (36.8)	
Size of enterprise (number of workers)			0.0114
<5	305 (21.6)	150 (25.5)	
5 ~ 9	342 (24.2)	154 (26.2)	
10 ~ 29	368 (26.1)	159 (27.0)	
≥30	397 (28.1)	125 (21.3)	
Working status			< 0.0001
Regular	851 (60.3)	249 (42.4)	
Temporary	166 (11.8)	102 (17.4)	
Daily	389 (27.6)	236 (40.1)	
Self-employed/employer	6 (0.4)	1 (0.1)	
Type of employment			0.0408
Full-time	1365 (96.7)	551 (94.7)	
Part-time	47 (3.3)	31 (5.3)	
Shift work			0.2778
Yes	167 (11.8)	59 (10.0)	
No	1245 (88.2)	529 (90.0)	
Employment contract			0.0003
Yes	725 (51.4)	250 (42.5)	
No	687 (48.7)	338 (57.5)	
Mean of working days per month	21.81 ± 5.09	21.79 ± 5.92	0.9463
Mean of working hours per day	9.40 ± 2.47	9.51 ± 2.50	0.3300

### Factors related to the physician and the employer

Regarding aspects of the physician, more respondents answered that they were provided a full explanation by the physician than those who indicated they did not receive sufficient explanation. In the non-RTW group, 17 % responded that they did not receive periodic recovery assessments during the medical care period. Further, 56.5 % of the respondents who believe that the duration of treatment was inadequate belonged to the non-RTW group. In contrast, in the RTW group were answered 10.9 and 40.8 % of these two questions negatively (each was statistically significant). And 26.6 % of the respondents in the RTW group and 22.3 % of those in the non-RTW group reported they had counseling about returning to work with the physician during the rehabilitation period; this is statistically significant (*p* = 0.0481). The subjects who had work capacity assessment and referral for vocational return were higher the RTW group than the non-RTW group with *p* =0.0481. In both the groups, most of the participants reported “satisfied: high” in terms of the satisfaction level with the consultation offered by the physician: 94.4 % of the RTW group and 92.5 % of the non-RTW group. Factors related to the physician and the employer of the RTW group and the non-RTW group are presented in Table 4.

**Table 4:** Factors related to the physician and the employer by economic activity status

	Return to work group	Non- return to work group	*p*-value
	*N* = 1412 (% = 70.6)	*N* = 588 (% = 29.4)	
The physician-related factors			
Detailed explanation of medical care			0.0821
Yes	1344 (95.2)	548 (93.2)	
No	68 (4.8)	40 (6.8)	
Regular assessment of recovery			0.0003
Yes	1258 (89.1)	488 (83.0)	
No	154 (10.9)	100 (17.0)	
Appropriateness of the treatment duration			< 0.0001
Yes	836 (59.2)	256 (43.5)	
No	576 (40.8)	332 (56.5)	
Counseling about returning to work			< 0.0001
Yes	143 (10.1)	27 (4.6)	
No	1269 (89.9)	561 (95.4)	
Work ability assessment and referral for vocational return			0.0481
Yes	375 (26.6)	131 (22.3)	
No	1037 (73.4)	457 (77.7)	
Satisfaction level with the physician			0.0480
Low	12 (0.8)	3 (0.5)	
Moderate	67 (4.8)	41 (7.0)	
High	1333 (94.4)	544 (92.5)	
The employer-related factors			
Maintenance of relation			< 0.0001
Yes	968 (68.6)	273 (46.4)	
No	444 (31.4)	315 (53.6)	
Provided convenience during recovery			0.0016
Yes	411 (70.9)	131 (22.3)	
No	1001 (29.1)	457 (77.7)	
Satisfaction level with employer			< 0.0001
Low	365 (25.8)	259 (44.1)	
Moderate	710 (50.3)	272 (46.3)	
High	337 (23.9)	57 (9.7)	

Regarding the employer-related factors, the maintenance of the relationship between the injured worker and the employer or related personnel after the industrial accident was positively reported by 68.6 % of the respondents in the RTW group and 46.4 % of those in the non-RTW group; this is statistically significant (*p* <0.0001). Regarding whether any facilities or convenience were offered by the employer during the convalescence period, 22.3 % of the participants in the non-RTW group and 70.9 % of the participants in the RTW group responded in the affirmative, which is statistically significant (*p* = 0.0016). Regarding overall satisfaction with the additional compensations from the employer, 44.1 % of the non-RTW group and 25.8 % of the RTW group responded “dissatisfied: low”; this is statistically significant (*p* <0.0001).

### Multivariable analysis for the RTW

Table 5 shows the ORs and 95 % CI of RTW in the multivariable logistic regression model. The results in Table 5 are estimated after adjusted for all other covariates excluding an interesting variant. Regarding the baseline characteristics, the RTW group revealed higher odds of 1.72 (95 % CI: 1.15–2.58) in male than female workers. The RTW group had a higher odds of 1.64 (95 % CI: 1.15–2.34) age group from 40 to 49 compared to the reference age group (under 40). The odds of RTW in age group more than 60 were 0.64 (95 % CI: 0.42–0.97) times higher than reference group. The odds of RTW in the first quartile of household income were higher than all other household income categories with statistical significant. The occupationally injured workers who were registered as disabled were less likely to RTW (ORs: 0.27; 95 % CI: 0.18–0.40) than the workers without disabilities. The odds of RTW group with a temporary job were 0.69 (95 % CI: 0.50–0.95) times lower than those of workers with a regular job.

**Table 5:** Odds Ratios and 95 % confidence interval for return to work in multivariate logistic regression analysis

	Odds ratio (95 % confidence interval)^a^
Baseline characteristics	
Sex	
Men	**1.72 (1.15–2.58)**
Women	1.00
Age (years)	
<40	1.00
40 ~ 49	**1.64 (1.15–2.34)**
50 ~ 59	1.38 (0.96–1.99)
≥60	**0.64 (0.42–0.97)**
Education	
Middle school	1.00
High school	1.12 (0.85–1.48)
College or higher education	1.48 (0.97–2.25)
Household income	
1st quartile	1.00
2nd quartile	**0.66 (0.50–0.87)**
3rd quartile	**0.54 (0.35–0.83)**
4th quartile	**0.51 (0.27–0.95)**
Smoking	
Current	1.01 (0.73–1.39)
Past	0.96 (0.66–1.38)
Never	1.00
Alcohol consumption	
Current	1.30 (0.96–1.76)
Past	**0.56 (0.36–0.87)**
Never	1.00
Registered as disabled	
Yes	**0.27 (0.18–0.40)**
No	1.00
Occupational characteristics	
Occupational category	
Non-manual	1.00
Manual	0.85 (0.54–1.34)
Other	1.16 (0.64–2.12)
Size of enterprise (number of workers)	
<5	0.74 (0.53–1.03)
5 ~ 9	0.82 (0.60–1.13)
10 ~ 29	0.75 (0.55–1.03)
≥30	1.00
Working status	
Regular	1.00
Temporary	**0.69 (0.50–0.95)**
Daily	0.79 (0.60–1.05)
Self-employed/employer	0.96 (0.10–8.83)
Type of employment	
Full-time	0.97 (0.57–1.64)
Part-time	1.00
Employment contract	
Yes	1.15 (0.92–1.45)
No	1.00
The physician-related factors	
Detailed explanation of medical care	
Yes	1.20 (0.95–1.52)
No	1.00
Regular assessment of recovery	
Yes	**1.51 (1.07–2.13)**
No	1.00
Appropriateness of the treatment duration	
Yes	**1.40 (1.12–1.77)**
No	1.00
Counseling about returning to work	
Yes	1.00 (0.74–1.36)
No	1.00
Work ability assessment and referral for vocational return	
Yes	**1.68 (1.05–2.69)**
No	1.00
Satisfaction level with the physician	
Low	1.00
Moderate	0.57 (0.14–2.34)
High	0.89 (0.23–3.49)
The employer-related factors	
Maintenance of relation	
Yes	**1.79 (1.41–2.26)**
No	1.00
Provided convenience during recovery	
Yes	0.95 (0.73–1.25)
No	1.00
Satisfaction level with employer	
Low	1.00
Moderate	**1.39 (1.09–1.78)**
High	**2.59 (1.78–3.75)**

Boldface text in the results column indicates a statistically significant *p*-value less than 0.05

^a^Statistical estimated from a multivariate logistic model that adjusted for all other covariates excluding an interesting variant

The ORs (95 % CI) of receiving periodic recovery assessment from the medical care institution were 1.51 (1.07–2.13) for the respondents belonging to the RTW group. The affirmative responses to the question whether the treatment duration is appropriate indicate that the ORs (95 % CI) are 1.40 (1.12–1.77) in the RTW group. Regarding to receiving a work ability assessment and a job reinstatement note, participants belonging to the RTW group were positively associated (ORs: 1.68; 95 % CI: 1.05–2.69).

The ORs (95 % CI) related to maintenance of the relation with the employer during the rehabilitation period are 1.79 (1.41–2.26) for the RTW group. The overall satisfaction level regarding the employer-related factors among the respondents who answered with a moderate level and a high level was higher than that of the respondents who answered with a low level (ORs: 1.39; 95 % CI: 1.09–1.78 and ORs: 2.59; 95 % CI: 1.78–3.75, respectively).

## Discussion

The study participants were divided into two groups according to their economic status: RTW and non-RTW participants. The RTW participants were found to have younger individuals, lower household income status, more non-disabled people, and less temporary working people compared to the non-RTW participants, with statistical significance in the multivariable logistic regression model. However, we could not find any statistical significance in terms of educational level, occupational category, size of the enterprise, type of employment, and whether the workers have an employment contract.

Prior studies mainly identified the characteristics of the vulnerable groups who cannot participate in RTW. For example, these studies identified women, the elderly, and low-income groups are being vulnerable in terms of RTW by analyzing the general characteristics and socio-economic characteristics of the occupationally injured workers [16]. The non-RTW participants had a higher disability grade or required long-term care [17]. Prior research reported that the RTW is difficult depending on the size of enterprise or in the context of certain kinds of business (electricity, gas and water supply) by analyzing the workplace that led to the industrial accident [18]. Some of these vulnerable characteristics related to the RTW were found in the present study.

A significant observation noted in this study is that the physician and the employer can play an important role in generating RTW for the occupationally injured worker. The physician is responsible for the convalescence, medical care services, and rehabilitation services offered under the workers’ compensation insurance policy in Korea (South).

The odds for RTW were found to be 1.51 times higher (95 % CI: 1.07–2.13) when the physician evaluated the recovery status regularly, even after adjusting all other covariates. With regard to the question about the appropriateness of the treatment duration, the odds of the RTW were 1.40 (95 % CI: 1.12–1.77) times greater than those of the non-RTW participants. The physician was found to be able to reduce the psychological insecurity caused by the industrial accident. Workers who meet with an industrial accident feel immense pain and a sense of loss because of the physical disability, and are known to suffer from psychological problems such as anxiety and depression [19]. Most of the occupationally injured workers complain about psychological problems, such as depression and fear, during the convalescence process following the industrial accident. Moreover, they feel stressed about the decline in physical ability or the loss of economic activity [1]. The physician can play an important role in eliminating such anxiety of these workers. While it is well-known that the physician’s sincere attitude and proper assessment of the injury can play a major role in reducing anxiety and stress, his/her role is extremely important in the convalescence and rehabilitation process as well [20, 21]. In addition, not only does the rehabilitation policy in the occupationally injured worker’s convalescence period have an effect on RTW, but it also enhances the quality of employment at the time RTW. Thus, the physician plays a pivotal role in the convalescence and rehabilitation services [4, 22].

The participants of the RTW group who receive work ability assessment and referral for vocational return have higher recovery than compared to the participants in the non-RTW group who do not receive these services. The workers’ compensation insurance policy generally focused on medical rehabilitation or financial compensation. There is increasing concern that vocational rehabilitation is underestimated compared to the medical or financial aspects [23, 24]. However, in the present study, the case where the participants received work capacity assessment and referral for vocational return demonstrated higher RTW compared to the case where the participants did not receive any of these benefits. Eventually, the plan to utilize the work capacity assessment and referral for vocational return in one of the services provided by KCOMWEL needs to be addressed. Further, the measures for resuming the RTW of the occupationally injured worker need to be considered.

The employer plays an important role with regard to the RTW of the occupationally injured worker. According to the findings of this study, however, providing an actual convenience or financial support such as paying the treatment cost during convalescence does not have a significant statistical impact on the RTW of the occupationally injured worker. When the employer or the HR personnel maintain a relationship with the occupationally injured worker in the form of visits or telephonic communication, this was found to have a higher impact on the RTW of the injured workers compared to the effect of providing substantial conveniences or facilities.

This result has a significant implication regarding the employers’ attitude. Avoiding the recognition of an industrial accident at work, delaying the reporting of a workplace accident, and delaying the payment of insurance fees for “injury on duty” are common ways used by employers to reduce the company’s burden in terms of providing compensation insurance for workers’ industrial injuries. The employer’s concern about financial burden in relation to industrial accidents is linked to the increase in insurance cost, inspections by the Ministry of Employment and Labor, and the terms of the Labor Occupational Safety and Health Act. Further, the requirement that additional compensation should be provided to an occupationally injured worker is reported to enhance the burden [25].

The result of this study indicates that non-monetary factors such as maintaining a relationship with the employer and overall satisfaction level with the employer have a high impact on RTW compared to the impact of monetary compensation and other conveniences provided during convalescence. The employer concerned about the financial burden such as an additional compensation for injured workers. But our results demonstrate that maintaining the relationship between the employer and the occupationally injured worker is more important factor to the RTW than financial compensation. Furthermore, the importance of non-monetary factors is clearly represented by the participants’ satisfaction level with the employer. The group that reported satisfaction related to the employer had about 2.59 times higher odds (95 % CI: 1.78–3.75) compared to the unsatisfied group; this was statistically significance. This represents the highest ORs reported in this analysis.

The importance of relationship between the employer and occupationally injured worker to return to work has been identified in prior studies [8, 26]. However, the previous study focuses on job retention. It is quite predictable that the interests of the employer/decision-makers regarding the original job have a positive impact on the job retention of the occupationally injured worker. However, this study shows that concern and care from the employer remain important in the overall perspective of RTW including job retention. The injured worker feels sorry for and guilty about his/her colleagues or family and is anxious about the future status after the industrial accident. Further, occupationally injured workers suffer more from psychiatric stress than from physical disorders [1, 27]. Therefore, maintaining a cordial relationship with the HR personnel and the employer can play an important role in eliminating regret, guilt, and anxiety. Since this contributes to the psychological stability of the occupationally injured worker during convalescence, this factor leads to better results during convalescence and rehabilitation [28].

The following limitations should be considered for improving the results of our study. This study used a cross-sectional design in which causal relationships between characteristics of individuals and the RTW remain unclear. A longitudinal study is necessary to reveal any specific cause-and-effect factors related to the RTW. However, studying the plan of the panel research related to the overall workers’ compensation insurance through the analysis of the Labor Welfare Corporation’s national panel survey has been attempted for the first time. Further, providing an overview of the entire industrial accident support system for an occupationally injured worker has been a significant contribution of this study. The research method required the preparation of detailed questionnaires for job retention, reemployment, self-employed, unpaid family worker, joblessness, and economical inactivity by identifying the current status of economic activity during the preparation of each questionnaire. The ensuing difficulty in comparing the data obtained is a shortcoming of this study because there is specific questionnaire for a distinct area for each group except the common questionnaires for some other areas. However, it is important to analyze the factors affecting the differences between the RTW and non-RTW groups using a common questionnaire.

## Conclusion

In summary, this study found that the RTW of an occupationally injured worker is affected by the physician and the employer. The roles and interests of the physician and the employer who carry out independent functions of the workers’ compensation insurance are particularly important, and they have greater implications on the efforts to promote the development of the current system of industrial accident insurance. Therefore, further research on the role of the physician and the employer in the service programs related to the workers’ compensation insurance would greatly help in the RTW of occupationally injured workers.
